# Navigating the Complexities of Carcinoid Heart Disease and Severe Coronary Artery Disease: A Case for Multidisciplinary Collaboration

**DOI:** 10.7759/cureus.99940

**Published:** 2025-12-23

**Authors:** Boris Arbit

**Affiliations:** 1 Cardiology, David Geffen School of Medicine at University of California, Los Angeles, Los Angeles, USA

**Keywords:** carcinoid heart disease, coronary artery disease, echocardiography, multidisciplinary approach, neuroendocrine tumor, right-sided heart failure

## Abstract

This case report presents a 71-year-old male with a complex cardiac pathology involving severe coronary artery disease (CAD) and carcinoid heart disease (CHD) secondary to metastatic neuroendocrine tumor (NET). The patient presented with symptoms of orthopnea, persistent lower extremity edema, and fatigue. Diagnostic investigations revealed a highly elevated coronary artery calcium score, echocardiographic evidence of right-sided heart involvement typical of CHD, and positron emission tomography/computed tomography (PET/CT) confirmation of metastatic NET. This case highlights the diagnostic challenges posed by the coexistence of CHD and CAD and emphasizes the need for a multidisciplinary approach to optimize patient management.

## Introduction

Carcinoid heart disease (CHD) is a rare but serious cardiac complication of neuroendocrine tumors (NETs), occurring in approximately 20-50% of patients with carcinoid syndrome [1,2]. The reported incidence of CHD in carcinoid syndrome varies widely because of differences in patient selection, diagnostic definitions, and screening intensity. Carcinoid syndrome primarily affects right-sided cardiac valves due to the deposition of fibrous plaques caused by vasoactive substances such as serotonin [3]. The left side of the heart is usually spared because the lungs contain monoamine oxidase, which efficiently degrades serotonin and related substances before they can reach the left side of the heart. The tricuspid and pulmonary valves are most commonly involved, leading to valvular dysfunction such as tricuspid regurgitation and pulmonary stenosis, which can progress to right-sided heart failure [4,5]. Symptoms often include fatigue, dyspnea, peripheral edema, and ascites, although these may initially be subtle [6].

The coexistence of CHD with other cardiac conditions like coronary artery disease (CAD) presents unique diagnostic and therapeutic challenges. CAD is characterized by atherosclerotic plaque buildup in coronary arteries and is a leading cause of morbidity in older adults [7]. The overlapping symptoms between CHD and CAD, such as fatigue and dyspnea, can obscure the clinical picture, necessitating comprehensive diagnostic evaluations.

Echocardiography remains the gold standard for diagnosing CHD. Specialized techniques such as three-dimensional echocardiography and strain imaging can enhance early detection of CHD changes by providing more precise anatomical and functional assessment than standard echocardiography, as supported by National Comprehensive Cancer Network guidelines and recent clinical evidence [8]. Positron emission tomography/computed tomography (PET/CT) scans and biochemical markers like 5-hydroxyindoleacetic acid (5-HIAA) are essential for confirming NETs and their metastases [9]. Management requires a multidisciplinary approach involving cardiology, oncology, and sometimes cardiothoracic surgery. Treatment strategies may include somatostatin analogs to control hormone secretion, diuretics for symptom relief, and valve replacement surgery in advanced cases [5].

This case report describes a patient with both CHD and severe CAD, emphasizing clinical difficulties as well as the need for collaborative care in managing complex cardiac presentations. The learning objectives are to highlight the importance of thorough diagnostic evaluation, multidisciplinary management, and individualized treatment planning in patients presenting with overlapping cardiac pathologies.

## Case presentation

A 71-year-old male with a known history of severe CAD presented to the Cardiology Clinic with progressive orthopnea, fatigue, persistent bilateral lower extremity swelling, and a recent five-pound weight gain. Additionally, he noted a gradual decline in exercise tolerance, which he initially attributed to aging. However, worsening symptoms prompted him to seek medical attention. The patient’s medical history included severe CAD, hyperlipidemia, diaphragmatic paralysis, and chronic venous insufficiency. The patient was a retired sports psychologist. He had recently stopped alcohol consumption and previously engaged in regular elliptical training, but had reduced his activity due to right leg pain. He denied any history of smoking or illicit drug use. On physical examination, cardiovascular assessment revealed bilateral lower extremity edema and jugular venous distension 9 cm above the sternal angle with prominent V waves. Auscultation identified a holosystolic murmur at the left lower sternal border consistent with tricuspid regurgitation. Respiratory examination revealed shortness of breath when supine and mild bibasilar crackles on auscultation. Appropriate diagnostic evaluation was then undertaken. A CT coronary calcium scan showed an elevated coronary artery calcium (CAC) score of 3951, placing the patient in the 90-100 percentile range for his age and gender. ECG demonstrated a normal sinus rhythm at 84 bpm without ST elevations or Q waves. An echocardiogram revealed preserved left ventricular ejection fraction (55-60%), mildly enlarged right ventricle with low normal systolic function, moderately dilated right atrium, restricted tricuspid valve motion with moderate regurgitation, mild-to-moderate pulmonary regurgitation, and mildly elevated right atrial pressure. A contrast bubble study with agitated saline did not show a patent foramen ovale (PFO), and no pericardial effusion was seen. The laboratory investigations showed increased levels of alkaline phosphatase, chromogranin-A, serotonin, and BNP (see Table 1). These parameters were markedly above their respective reference ranges, indicating significant deviations from normal values.

**Table 1:** Laboratory investigations showing increased levels of alkaline phosphatase, chromogranin-A, serotonin, and BNP. BNP: B-type natriuretic peptide

Laboratory Parameter	Value	Reference Range
Alkaline Phosphatase	183 u/L	34-104 u/L
Chromogranin-A	29,820 ng/mL	0-187 ng/mL
Serotonin	2,635 ng/mL	50-220 ng/mL
BNP	202 pg/mL	<100 pg/mL

A PET/CT scan conducted during the same period identified avid DOTATATE uptake within the stomach (suggesting the primary tumor site) as well as multiple hepatic lesions with similar uptake patterns consistent with metastatic NET involvement. Treatment with somatostatin analogs was initiated. After several weeks, the patient's clinical course deteriorated with progressive shortness of breath, lower extremity edema, and orthopnea. When efforts of conservative management with diuresis proved futile, the decision was made to proceed with tricuspid and pulmonic valve replacement as well as coronary artery bypass graft surgery. Carcinoid crisis, a potentially life-threatening complication during surgery, marked by the sudden release of vasoactive substances (causing profound hemodynamic instability), was discussed. Given the findings of recent meta-analyses and consensus guidelines, prophylactic octreotide was not administered [10]. Intraoperative transesophageal echocardiogram confirmed the presence of immobile and incompetent tricuspid and pulmonary arteries (see Videos 1, 2).

**Video 1:** Transesophageal echocardiogram. Short-axis view of the tricuspid valve. The leaflets appear significantly thickened and rigid due to carcinoid plaque deposition.

**Video 2:** Three-dimensional transesophageal echocardiogram. Real-time 3D echocardiogram showing pulmonary valve cusps that are diffusely thickened, shortened, and retracted.

**Figure 1:**
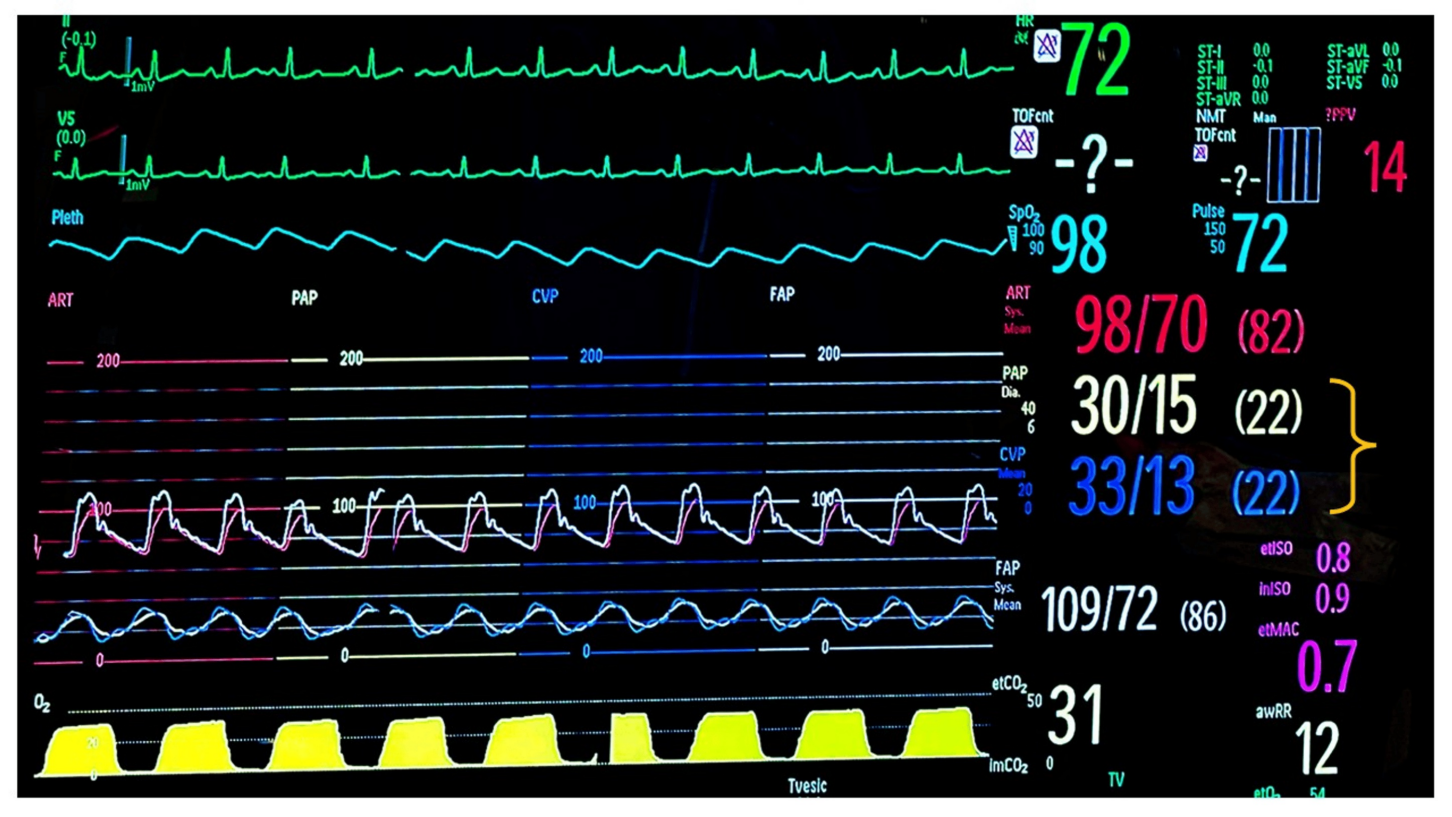
Intraoperative hemodynamics. Intraoperative hemodynamics show pulmonary artery pressure and central venous pressure to be the same (yellow bracket), indicating right ventricular failure and severe tricuspid regurgitation.

Intraoperative pulmonary artery catheter findings showed a central venous pressure (CVP) of 22 mmHg and pulmonary artery pressure (PAP) of 30/15 mmHg. These values corresponded to pulmonary artery pulsatility index (PAPi) of 0.68 (PAPi < 0.9 often indicates possible right ventricular failure and increased risk of mechanical support and mortality) (Figure 1). Due to severe right ventricular dysfunction and subsequent cardiogenic shock, extracorporeal membrane oxygenation (ECMO) was required postoperatively. Following this, the patient developed fulminant liver failure with severe coagulopathy and bleeding. Shortly, the patient developed multisystem organ failure requiring mechanical ventilation, hemodialysis, and antibiotics for treatment of sepsis. In light of the patient's critical condition and rapid deterioration, a decision was made to turn to comfort measures, and the patient soon passed.

## Discussion

This case presents a complex interplay between severe CAD and CHD secondary to metastatic NET. The patient's symptoms - orthopnea, persistent lower extremity edema, fatigue - and echocardiographic findings strongly suggested CHD as the predominant pathology despite coexisting CAD.

Echocardiographic findings typical of CHD included restricted tricuspid valve motion with moderate regurgitation, right ventricular enlargement with low-normal systolic function, pulmonary regurgitation, and elevated right atrial pressure [3,4]. These findings were corroborated by PET/CT imaging showing metastatic NET involvement in the liver. The characteristic valvular lesions observed in this patient align with the pathophysiological mechanism of CHD, wherein vasoactive substances, primarily serotonin released by NET metastases, escape hepatic inactivation and induce fibrotic changes in cardiac valves [5]. The predominant involvement of right-sided valves occurs because pulmonary circulation metabolizes these vasoactive substances before they can affect the left heart. Importantly, comprehensive echocardiographic assessment should include careful evaluation for PFO, as its presence can allow these vasoactive substances to bypass pulmonary metabolism and directly access the systemic circulation, potentially causing left-sided valve involvement. This has significant implications for disease staging, surgical planning, and overall prognosis [9,11]. In our patient, the absence of left-sided valve disease suggested no right-to-left shunting, which was further confirmed by a negative bubble study.

The coexistence of CAD further complicated this case. While CAD can contribute to symptoms such as fatigue or reduced exercise tolerance [7], the predominant right-sided heart involvement pointed more towards CHD as the primary driver of clinical deterioration.

Our patient had a long-standing history of diaphragmatic paralysis. This was important to consider in his care, as diaphragmatic paralysis can significantly complicate the diagnosis of severe CAD and CHD because it is an underrecognized cause of dyspnea and exercise intolerance, symptoms that overlap with both cardiac conditions. Distinguishing the relative contributions of each pathology to the patient's symptomatology presented a significant clinical challenge. The patient's elevated N-terminal pro-B-type natriuretic peptide (NT-proBNP) levels, while potentially attributable to either condition, were more consistent with the degree of right heart dysfunction observed, further supporting CHD as the principal cardiac pathology.

Management of this patient required close collaboration between specialties. Cardiologists played a central role in diagnosing CHD using echocardiography; oncologists guided systemic therapy for NET; and cardiothoracic surgeons were consulted regarding potential valve replacement surgery [6,8]. Multimodal imaging was critical for accurate diagnosis and treatment planning [12].

This case report is limited by its single-patient nature, which restricts the generalizability of the findings. The coexistence of CHD and severe CAD presents overlapping clinical and hemodynamic features, making it challenging to delineate the individual contribution of each condition to the patient’s presentation. Additionally, the temporal relationship between the two diseases remains uncertain. Management decisions, including combined valve replacement and coronary artery bypass surgery, were based on multidisciplinary clinical judgment rather than established guidelines, reflecting the absence of standardized treatment pathways for such complex cases. Finally, the advanced stage of disease and lack of long-term follow-up data limit assessment of therapeutic efficacy and outcomes.

## Conclusions

This case highlights the diagnostic challenges associated with the coexistence of CHD and severe CAD. It emphasizes the need for a high index of suspicion for CHD in patients with NETs who present with right-sided heart failure symptoms. Comprehensive assessment with echocardiography and PET/CT imaging is essential for accurate characterization of both conditions.

Optimal management requires close collaboration among cardiology, oncology, radiology, and cardiothoracic surgery teams to address both cardiac pathologies in a coordinated manner. Such multidisciplinary involvement is critical not only across specialties but also throughout all phases of care - from diagnosis and preoperative planning to intraoperative management and postoperative follow-up. Early recognition and timely intervention remain key to improving outcomes in these complex, multifaceted cases.
